# Time trends in mechanical thrombectomy (2017–2021): do real-world data reflect advances in evidence?

**DOI:** 10.3389/fneur.2024.1517276

**Published:** 2025-02-11

**Authors:** Christoph Riegler, Viktoria Rücker, Regina von Rennenberg, Kerstin Bollweg, Bastian Cheng, Anna C. Alegiani, Fabian Flottmann, Marlena Schnieder, Marielle Ernst, Waltraud Pfeilschifter, Christoffer Kraemer, Ruben Mühl-Benninghaus, Steffen Tiedt, Lars Kellert, Hanna Zimmermann, Felix J. Bode, Gabor C. Petzold, Franziska Dorn, Jörg Berrouschot, Albrecht Bormann, Kathleen Bernkopf, Silke Wunderlich, Tobias Boeckh-Behrens, Martina Petersen, Lars Udo Krause, Stephan Lowens, Heinrich J. Audebert, Eberhard Siebert, Peter U. Heuschmann, Christian H. Nolte

**Affiliations:** ^1^Department of Neurology, Charité – Universitätsmedizin Berlin, Corporate Member of Freie Universität Berlin and Humboldt Universität zu Berlin, Berlin, Germany; ^2^Center for Stroke Research Berlin (CSB), Charité – Universitätsmedizin Berlin, Berlin, Germany; ^3^Institute of Clinical Epidemiology and Biometry, Julius-Maximilians-Universität (JMU) Würzburg, Würzburg, Germany; ^4^Department of Neurology, University Medical Center Hamburg-Eppendorf, Hamburg, Germany; ^5^Department of Neuroradiology, University Medical Center Hamburg-Eppendorf, Hamburg, Germany; ^6^Department of Neurology, University Medical Center Göttingen, Göttingen, Germany; ^7^Institute of Diagnostic and Interventional Neuroradiology, University Medical Center Göttingen (UMG), Georg-August-University Göttingen, Göttingen, Germany; ^8^Department of Neurology and Clinical Neurophysiology, Städtisches Klinikum Lüneburg, Lüneburg, Germany; ^9^Department of Neurology, Centre of Neurology and Neurosurgery, Goethe University, Frankfurt, Germany; ^10^University Hospital Frankfurt, Frankfurt, Germany; ^11^Department of Radiology, Städtisches Klinikum Lüneburg, Lüneburg, Germany; ^12^Institute for Stroke and Dementia Research, University Hospital Ludwig-Maximilian University, Munich, Germany; ^13^Department of Neurology, University Hospital, LMU Munich, Munich, Germany; ^14^Institute for Neuroradiology, Ludwig-Maximilians-University (LMU) Munich, Munich, Germany; ^15^Department of Vascular Neurology, University Hospital Bonn, Bonn, Germany; ^16^Department of Neurology, University Hospital Bonn, Bonn, Germany; ^17^Department of Neuroradiology, University Hospital, Bonn, Germany; ^18^Klinik für Neurologie, Klinikum Altenburger Land, Altenburg, Germany; ^19^Klinik für Radiologie, Interventionsradiologie und Neuroradiologie, Klinikum Altenburger Land, Altenburg, Germany; ^20^Department of Neurology, School of Medicine Klinikum Rechts der Isar, Technical University of Munich, Munich, Germany; ^21^Department of Diagnostic and Interventional Neuroradiology, Klinikum Rechts der Isar, Technical University Munich, Munich, Germany; ^22^Department of Neurology, Klinikum Osnabrück, Osnabrück, Germany; ^23^Department of Radiology, Klinikum Osnabrück, Osnabrück, Germany; ^24^Department of Neuroradiology, Charité - Universitätsmedizin Berlin, Berlin, Germany, Corporate Member of Freie Universität Berlin and Humboldt-Universität zu Berlin, Berlin, Germany; ^25^Institute of Medical Data Science, University Hospital Würzburg, Würzburg, Germany; ^26^Clinical Trial Center Würzburg, University Hospital Würzburg, Würzburg, Germany; ^27^Berlin Institute of Health (BIH) at Charité – Universitätsmedizin Berlin, Berlin, Germany; ^28^Deutsches Zentrum für Herz-Kreislaufforschung DZHK, Berlin, Germany

**Keywords:** ischaemic stroke, endovascular therapy, mechanical thrombectomy, time trends, real world data

## Abstract

**Background:**

In recent years, we have witnessed a continuous, evidence-based expansion of indications for endovascular therapy (EVT) in the treatment of ischaemic stroke, driven by advancements in extended time windows and target vessel occlusion. Our study aimed to evaluate the temporal changes in patients’ characteristics, treatment, and outcomes in clinical practice.

**Methods:**

We used data from the German Stroke Registry, a large national multicentre prospective registry, which includes all patients receiving EVT for ischaemic stroke at its participating centers. We analysed baseline factors, treatment details, and clinical outcomes [Modified Rankin Scale (mRS) at 3 months] over a 5-year period (2017–2021).

**Results:**

We included 6,251 patients from eight centres. Over time, the characteristics of patients undergoing EVT changed in several aspects (2017 vs. 2021). Patients became older (median age from 76 [IQR: 65–82] to 77 [65–84 years]; p_trend_ = 0.02), and less severely affected (NIHSS from 15 [11–19] to 13 [8–18]; p_trend_ <0.001). There was an increase in patients treated more than 6 h after last seen well (22.0% to 28.3%; p_trend_<0.001), and more patients were treated for medium vessel occlusion (16.1% to 28.1%; p_trend_<0.001). The use of intravenous thrombolysis decreased (52.4% to 40.4%; p_trend_<0.01). Good functional outcome declined (percentage of patients with mRS ≤ 2 from 36.0 to 34.9%; aOR 0.94 per year [0.89–0.99]), while mortality at 3 months increased from 25.3% in 2017 to 34.7% in 2021; aOR 1.13 per year [1.07–1.19].

**Conclusion:**

Between 2017 and 2021, there were significant shifts in the demographic and clinical profiles of patients undergoing EVT, along with an expansion in EVT indications. Despite these patients presenting with less severe stroke symptoms, improvements in functional outcomes were not observed, and mortality rates increased. These trends may reflect willingness to treat patients with more severe underlying health conditions.

## Introduction

In 2015, several trials demonstrated the efficacy of endovascular treatment (EVT) for patients with large vessel occlusion (LVO) stroke (1). Initially, guidelines recommended performing EVT within 6 h of symptom onset (2). However, further evidence from randomised controlled trials (RCTs) extended this time window, demonstrating that EVT can be beneficial for up to 24 h after the last known well time in selected patients (3, 4). The concept of omitting intravenous thrombolysis (IVT) before EVT has been a topic of considerable debate; however, the available evidence has not been found to be sufficient to justify withholding IVT in eligible patients (5–7). Only since 2022 have guidelines explicitly recommended EVT together with IVT over EVT alone (5, 6). While EVT for LVO is widely implemented, the risks and benefits of EVT for medium vessel occlusions (MeVO) are yet to be proven, with several RCTs ongoing to produce evidence (8). Naturally, growing evidence derived from RCTs is transferred to clinical practise. Nevertheless, it is well-known that patient characteristics in randomised trials differ substantially from populations in clinical practise (9, 10). Outcomes in real-world cohorts tend to be worse (11), and higher rates of withdrawal of care may play a crucial role in this difference in outcomes (12, 13). Consequently, class 1 evidence cannot be transferred to clinical practise without restriction (9).

Aiming to investigate how recent evidence on EVT translates into clinical practise, we analysed changes in patients’ characteristics and clinical and technical outcomes over a 5-year period in a large cohort of patients from clinical routine in Germany.

## Methods

### Study population

**Figure 1:**
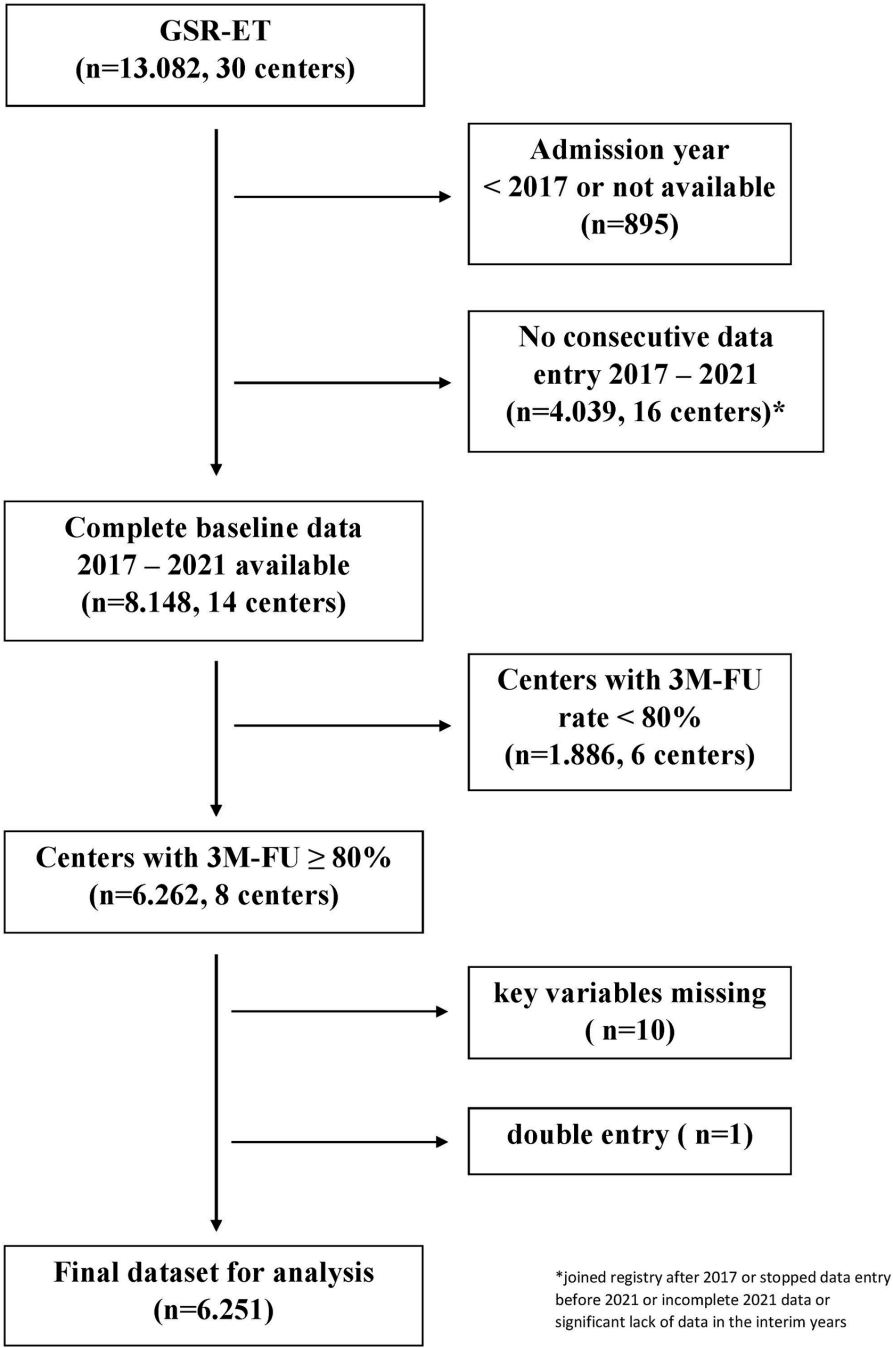
Selection of study centres from the GSR-ET cohort.

This study used data from the German Stroke Registry (GSR-ET), a national, multicenter, prospective observational registry that has been described in detail before (14, 15). The ongoing GSR-ET includes all consecutive individuals who receive EVT for ischaemic stroke in its participating centres (14). A systematic follow-up on functional status 3 months after stroke via Modified Rankin Scale (mRS) is regularly performed (16). We defined five consecutive years (2017–2021) as the period of interest. A structured questionnaire was conducted to assess centre-specific data quality and completeness, evaluating absolute numbers of EVT procedures and ischaemic stroke patients per year (via the hospitals’ quality assurance and controlling department). All centres with consecutive data entry over the specified 5-year period and a three-month follow-up rate of at least 80% were included in the analysis. Detailed information on the in-and exclusion of centres with respective patient numbers is depicted in Figure 1. Time trends were assessed in relation to baseline variables, procedural aspects (IVT, time-to-treatment, vessel occlusion target), and technical and functional outcomes from 2017 to 2021.

### Variables

Stroke severity was assessed using the National Institutes of Health Stroke Scale (NIHSS).

Early ischaemic changes on admission imaging were classified using ASPECTS (Alberta Stroke Program Early CT Score). Functional independence at 3 months (mRS ≤ 2; *good outcome*) was defined as the primary clinical outcome. As secondary outcome, *fair outcome* (dependency with unassisted ambulation, mRS ≤ 3 at 3 months) and disability assessed through mRS shift analysis were selected.

As short-term clinical outcomes, we assessed *early neurologic improvement* defined as a decrease of at least four points on the NIHSS or reaching an NIHSS score of 0 at 24 h. *Early neurologic deterioration* was defined as an increase in the NIHSS of at least four points between admission and assessment at 24 h. The *modified Thrombolysis in Cerebral Infarction scale* (mTICI) was applied to evaluate technical success at the end of the procedure. *Successful recanalisation* was defined as mTICI 2b or 3, and *complete recanalisation* as mTICI 3. Vasospasms, periprocedural clot migration, and dissection/perforation were assessed as procedural complications of interest. As clinical safety outcomes, in-hospital death, death within 3 months, any intracranial haemorrhage (ICH), and symptomatic ICH (sICH) were selected, with sICH as defined by ECASS-II criteria (any ICH with NIHSS worsening of four or more points).

### Statistical analysis

Continuous baseline variables and treatment times are presented as median [interquartile range; IQR], and dichotomous variables as absolute numbers and percentages. Time trends for linear and ordinal variables were analysed using the Jonckheere-Terpstra test (p for trend) (17). The Cochran-Armitage test (p-for-trend) was applied to assess trends for dichotomous variables (18). Functional disability at 3 months (mRS shift) was calculated using mixed model ordinal regression with *year of treatment* as a linear factor (estimated effects per +1 year). Adjustments were made for all potential confounders (inclusion model), namely age, sex, NIHSS at baseline, pre-stroke dependency (premorbid mRS > 2), intravenous thrombolysis, time from last-seen-well (or symptom onset) to arrival at the EVT hospital, diabetes mellitus, smoking status, hyperlipidaemia, arterial hypertension, atrial fibrillation, pre-stroke antiplatelets, pre-stroke anticoagulation, vessel occlusion site (MeVo vs. LVO), successful recanalisation (mTICI 2b/3 vs. mTICI <2b), and centre of treatment (as a random effect). In the ordinal (shift) model, common ORs < 1 indicate a worse outcome (higher disability), while ORs > 1 indicate reduced disability during the observed time period (per year, respectively).

Binary logistic mixed model analyses adjusting for the above-mentioned factors were conducted to assess the impact of *year of treatment* on binary clinical outcomes and safety variables. The following sensitivity analyses were conducted for all clinical and safety outcomes: (1) stratifying the patient population by time from last seen well (LSW) to hospital arrival (≤6 h vs. >6 h); (2) stratifying patients by pre-stroke disability (mRS >2; yes/no); (3) stratifying patients according to the COVID-19 pandemic into pre-pandemic (2017–2019) and pandemic (2020–2021) eras. Odds ratios (ORs) for successful recanalisation and complete recanalisation were adjusted for intravenous thrombolysis, stroke aetiology [large-artery atherosclerosis (LAA) vs. else], occlusion site (isolated extracranial vs. LVO vs. MeVO), and centre as a random effect. *Number of passes* was included in the model for treatment adverse events. All analyses were conducted using IBM SPSS Statistics for Windows, Version 27.0.0.0, Armonk, NY: IBM Corp.

### Informed consent and ethics approval

The GSR-ET registry was centrally approved by the Ethics Committee of the Ludwig-Maximilians University LMU, Munich (689–15) (14). Informed consent was not mandatory in accordance with local rules and regulations. For quality assurance reasons, data sampling from patients undergoing EVT is mandated by federal law. Thus, selection bias through lack of informed consent was minimised (16).

## Results

### Baseline demographics

After applying the pre-specified selection criteria to the GSR-ET dataset, the final study sample consisted of 6,251 patients from eight centres (five university hospitals and three public hospitals) (for detailed patient numbers and centre selection, see Figure 1).

Between 2017 and 2021, a slight increase in median age from 76 (IQR: 65–82) to 77 (65–84), p_trend_ < 0.02, was found. Sex distribution and pre-stroke dependency (pre-mRS > 2) did not change over the 5-year period. Median stroke severity decreased from 15 (11–19) in 2017 to 13 (8–18) in 2021, p_trend_ < 0.001. Regarding cardiovascular risk factors, relevant increases in the prevalence of dyslipidaemia (42.2% to 50.4%, p_trend_ < 0.001) and current smoking (12.5% to 19.0% p_trend_ < 0.001) were observed. While no increase in the prevalence of atrial fibrillation was identified, the proportion of patients taking anticoagulants at admission increased from 19.1 to 25.1% (p_trend_ < 0.001). All baseline variables are reported in Table 1.

**Table 1:** Baseline demographics, imaging, and treatment characteristics.

	2017 (*n* = 902)^¥^	2018 (*n* = 1300)	2019 (*n* = 1269)	2020 (*n* = 1383)	2021 (*n* = 1397)	p for trend*
Age (years)–median (IQR)	76 (65–82)	77 (67–83)	78 (67–84)	77 (67–83)	77 (65–84)	**0.02**
Female – n (%)	466/902 **(51.7)**	665/1300 **(51.2)**	642/1269 **(50.6)**	733/1383 **(53.0)**	739/1397 **(52.9)**	0.29
Pre-stroke Dependency (mRS > 2) – n (%)	105/894 **(11.7)**	195/1293 **(15.1)**	169/1251 **(13.5)**	196/1368 **(14.3)**	179/1378 **(13.0)**	0.86
NIHSS at admission – median (IQR)	15 (11–19)	14 (9–19)	14 (9–19)	14 (8–18)	13 (8–18)	**<0.001**
Hypertension – n (%)	668/902 **(74.1)**	1031/1300 **(79.3)**	998/1269 **(78.6)**	1053/1383 **(76.1)**	1017/1397 **(72.8)**	**0.03**
Diabetes mellitus – n (%)	206/902 **(22.8)**	269/1300 **(20.7)**	288/1269 **(22.7)**	304/1383 **(22.0)**	320/1397 **(22.9)**	0.58
Dyslipidemia – n (%)	381/902 **(42.2)**	604/1300 **(46.5)**	604/1269 **(47.6)**	681/1383 **(49.2)**	702/1397 **(50.4)**	**<0.001**
Atrial fibrillation – n (%)	379/902 **(42.0)**	592/1300 **(45.5)**	533/1269 **(42.0)**	552/1383 **(39.9)**	565/1397 **(40.4)**	**0.03**
Current smoking – n (%)	113/902 **(12.5)**	190/1300 **(14.6)**	186/1269 **(14.7)**	215/1383 **(15.5)**	266/1397 **(19.0)**	**<0.001**
Antiplatelets – n (%)	261/902 **(28.9)**	394/1300 **(30.3)**	337/1269 **(26.6)**	388/1383 **(28.1)**	378/1397 **(27.1)**	0.13
Anticoagulation^1^ – n (%)	172/902 **(19.1)**	281/1300 **(21.6)**	317/1269 **(25.0)**	301/1383 **(21.8)**	350/1397 **(25.1)**	**<0.01**
Stroke aetiology at discharge – n (%) - Cardioembolic - Large-artery-atherosclerosis - Dissection - Other determined - Undetermined	470/902 **(52.1)** 213/902 **(23.6)** 23/902 **(2.5)** 27/902 **(3.0)** 169/902 **(18.7)**	679/1300 **(52.2)** 313/1300 **(23.3)** 22/1300 **(1.7)** 50/1300 **(3.8)** 236/1300 **(18.2)**	676/1269 **(53.3)** 324/1269 **(25.6)** 17/1269 **(1.3)** 28/1269 **(2.2)** 224/1269 **(17.7)**	673/1383 **(48.7)** 373/1383 **(26.0)** 21/1383 **(1.5)** 55/1383 **(4.0)** 261/1383 **(18.9)**	710/1397 **(50.8)** 339/1397 **(22.8)** 22/1397 **(1.5)** 61/1397 **(4.4)** 266/1397 **(19.0)**	0.16 0.35 0.10 0.08 0.62
MRI at admission – n (%)	60/866 **(6.9)**	60/1251 **(4.8)**	48/1204 **(4.0)**	74/1290 **(5.7)**	56/1266 **(4.4)**	0.11
Perfusion-based imaging – n (%)	354/866 **(40.9)**	587/1251 **(46.9)**	667/1204 **(55.4)**	712/1290 **(55.2)**	748/1266 **(59.1)**	**<0.001**
Occlusion site – n (%) - Isolated extracranial - LVO - MeVO	37/898 **(4.1)** 716/898 **(79.7)** 145/898 **(16.1)**	44/1298 **(3.4)** 972/1298 **(74.9)** 282/1298 **(21.7)**	60/1267 **(4.7)** 917/1267 **(72.4)** 290/1267 **(22.9)**	56/1375 **(3.4)** 914/1375 **(66.5)** 405/1375 **(29.5)**	45/1329 **(3.9)** 910/1329 **(68.5)** 374/1329 **(28.1)**	**<0.001**
Internal carotid artery stenosis >70% - n (%)	125/902 **(13.9)**	154/1300 **(11.8)**	181/1269 **(14.3)**	185/1383 **(13.4)**	144/1397 **(10.3)**	0.06
ASPECTS – median (IQR) (missing n = 1402)	9 (7–10)	9 (7–10)	9 (7–10)	9 (7–10)	9 (7–10)	0.32
Witnessed onset of stroke – n (%)	524/902 **(58.1)**	698/1300 **(53.7)**	679/1269 **(53.3)**	731/1383 **(52.9)**	738/1397 **(52.8)**	**0.03**
Transferred from primary hospital – n (%)	373/902 **(41.4)**	611/1300 **(47.0)**	565/1269 **(44.5)**	619/1383 **(44.8)**	523/1397 **(37.4)**	**<0.01**
Symptom onset to admission (minutes) – median (IQR)	134 (61–206)	130 (60–211)	124 (58–207)	134 (64–223)	132 (66–215)	0.05
Last-seen well to admission (minutes) – median (IQR)	360 (207–722)	361 (215–680)	403 (213–720)	420 (226–785)	427 (226–749)	**0.01**
Symptom onset or last-seen well to admission ≥ 6 h – n (%)	181/822 (**22.0**)	288/1172 (24.6)	294/1146 (25.7)	354/1257 (28.2)	340/1203 (28.3)	**<0.001**
General anaesthesia – n (%)	541/879 **(61.5)**	824/1270 **(64.9)**	832/1232 **(67.5)**	944/1369 **(69.0)**	962/1303 **(73.8)**	**<0.001**
Admission to groin puncture (minutes) – median (IQR)	64 (40–98)	60 (38–88)	65 (42–91)	67 (45–98)	71 (49–97)	**<0.001**
Groin to flow restoration (minutes) – median (IQR)	41 (25–68)	38 (24–64)	42 (27–66)	39 (26–63)	45 (28–70)	**<0.001**
Number of passes – median (IQR)	2 (1–3)	2 (1–3)	2 (1–3)	2 (1–3)	2 (1–3)	0.20
Intravenous thrombolysis – n (%)	473/902 **(52.4)**	619/1300 **(47.6)**	562/1269 **(44.3)**	603/1383 **(43.6)**	565/1397 **(40.4)**	**<0.01**
Admission to thrombolysis (minutes) median (IQR)	29 (24–41)	28 (21–38)	34 (24–48)	31 (23–44)	33 (24–45)	**<0.01**

^¥^One centre started in Q3/2017 and one centre in Q4 2017. *Jonckheere-Terpstra for linear + ordinal variables, Cochran-Armitage for categorical and binary variables. **Concurrent occlusion of the extracranial internal carotid artery and middle or anterior cerebral artery; mRS, Modified Rankin scale; NIHSS, National Institutes of Health Stroke Scale; MRI, Magnetic Resonance Imaging; Isolated Extracranial, Extracranial internal carotid artery (ICA); vertebral artery; LVO, large vessel occlusion, including intracranial ICA, middle cerebral artery (MCA) M1 segment, and basilar artery; MeVo, medium vessel occlusion, including the MCA M2 segment, anterior cerebral artery, and posterior cerebral artery. Percentages and *p*-values <0.05 marked bold.

### Procedural variables

In patients with known onset of stroke, the median time from symptom onset to hospital arrival was stable at approximately 2 h over the whole 5-year period. However, in patients with unwitnessed onset of symptoms, the time from last seen well to hospital arrival increased from 360 (207–722) min in 2017 to 427 (226–749) min in 2021. The proportion of patients arriving more than 6 h after symptom onset or last-seen well (LSW) rose from 22.0 to 28.3% (p_trend_ < 0.001, respectively). The use of perfusion-based imaging at baseline increased continuously from 40.9 to 59.1% (p_trend_ < 0.001), while rates of thrombolysis decreased from 52.4 to 40.4% (p_trend_ < 0.001). There was a shift towards medium vessel occlusions (MeVO) as EVT targets: MeVO defined as occlusion of the anterior cerebral artery (ACA), posterior cerebral artery (PCA), or M2 segment of the middle cerebral artery (MCA) increased from 16.1 (2017) to 28.1% (2021) of all EVT patients. While in 2017, 61.5% of patients had general anaesthesia during the procedure, numbers increased to 73.8% in 2021 (p_trend_ < 0.001). Both the time from hospital arrival to groin puncture and the time from groin puncture to flow restoration increased during the 5-year period (door-to-groin: from 64 (40–98) min to 71 (49–97) min; groin-to-reperfusion: from 41 (25–68) in 2017 to 45 (28–70) in 2021, p_trend_ < 0.001). All procedural variables are reported in Table 1.

### Technical and clinical outcomes

For technical outcomes, improved rates of successful recanalisation (mTICI 2b/3 from 83.9 to 85.5%; aOR 1.07 [1.01–1.13] per +1 year, *p* = 0.01), successful recanalisation at first pass (from 35.8 to 41.6%; aOR 1.06 [1.01–1.10] per +1 year, *p* < 0.01), and complete recanalisation (mTICI 3 from 46.7% to. 54.2%; aOR 1.07 [1.03–1.11] per +1 year, *p* < 0.001) were observed.

While rates of early neurologic improvement (ENI) decreased from 43.5 to 38.1% (aOR 0.95 [0.93–0.995], *p* = 0.03), the frequency of early neurologic deterioration (END) increased over time, with 19.9% experiencing END in 2017 and 23.3% in 2021 (aOR 1.08 [1.02–1.14] per +1 year, *p* < 0.01).

**Figure 2:**
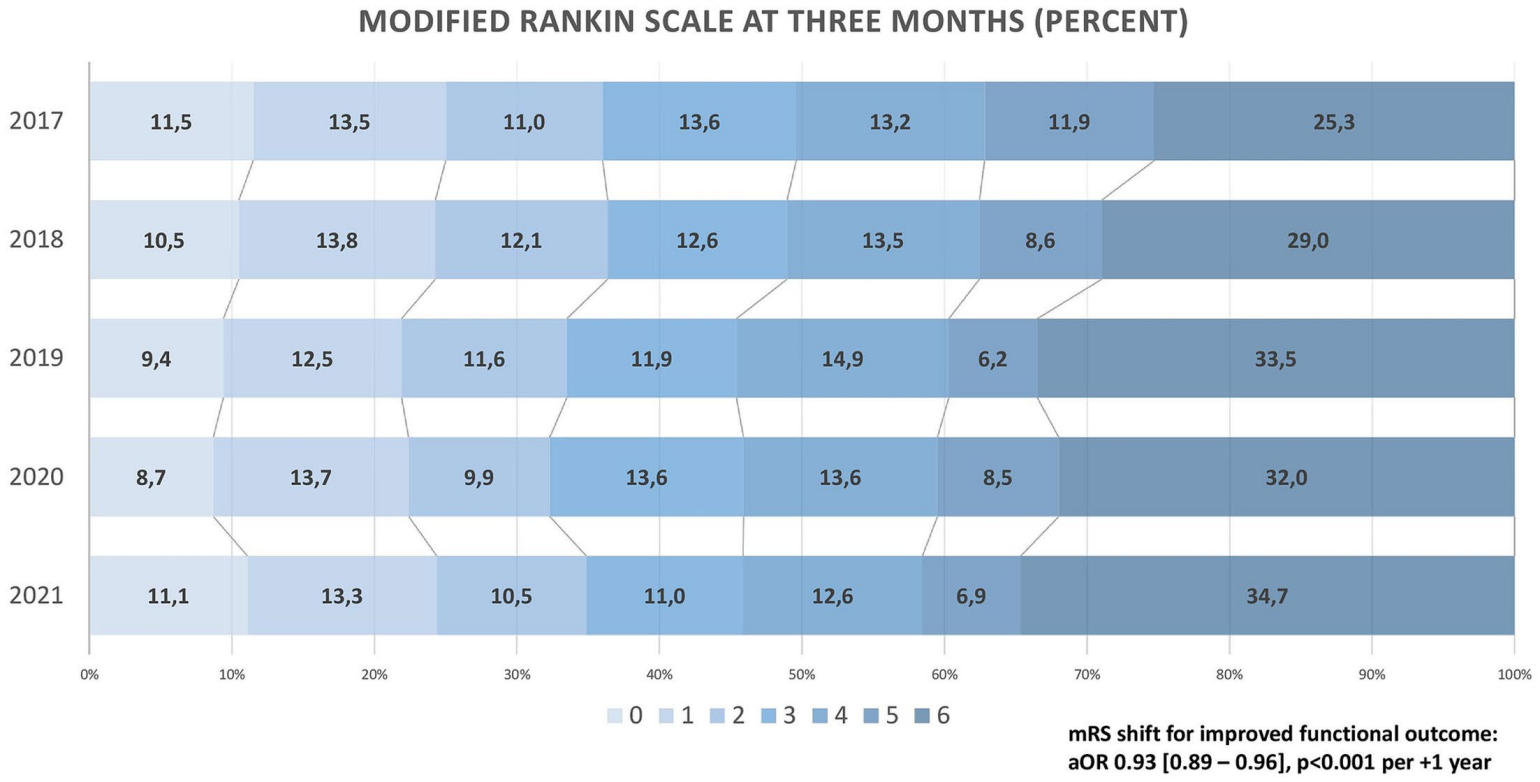
Clinical outcome on the Modified Rankin Scale (2017–2021).

Good outcome at 3 months (mRS ≤ 2) decreased from 36.0 to 34.9% (aOR 0.94 [0.89–0.99] per +1 year, *p* = 0.03). Rates of fair outcome (mRS ≤ 3) decreased from 49.7 to 45.8% (aOR 0.92 [0.88–0.97] per +1 year, *p* = 0.02), and as depicted in Figure 2, clinical outcome measured via mRS shift worsened over the 5-year period (adjusted common OR for reduced disability on the mRS 0.95 [0.92–0.99] per +1 year, p < 0.01). Despite stable rates of sICH (4.4% in 2017 vs. 4.4% in 2021), an increase in in-hospital mortality (14.1% vs. 20.8%; aOR 1.11 [1.04–1.17] per +1 year, *p* < 0.01) and mortality at 3 months (25.3% vs. 34.7%; aOR 1.13 [1.07–1.19] per +1 year, *p* < 0.001) was found. All technical and clinical outcomes are presented in Table 2.

**Table 2:** Clinical, technical, and safety outcomes.

	2017 (*n* = 902)*	2018 (*n* = 1300)	2019 (*n* = 1269)	2020 (*n* = 1383)	2021 (*n* = 1397)	aOR [95%-CI] per + 1 year	*p*
mRS ≤ 2 at d90 – n (%)	309/858 **(36.0)**	449/1235 **(36.4)**	379/1132 **(33.5)**	424/1313 **(32.3)**	426/1222 **(34.9)**	0.94 [0.89–0.99]^**1**^	**0.03**
mRS ≤ 3 at d90 – n (%)	426/858 **(49.7)**	604/1235 **(48.9)**	514/1132 **(45.4)**	603/1313 **(45.9)**	560/1222 **(45.8)**	0.92 [0.88–0.97]^**1**^	**<0.01**
Mortality at d90 – n (%)	217/858 **(25.3)**	358/1235 **(29.0)**	379/1132 **(33.5)**	420/1313 **(32.0)**	424/1222 **(34.7)**	1.13 [1.07–1.19]^**1**^	**<0.001**
mRS at d90 – median (IQR)	4 (1–6)	4 (2–6)	4 (2–6)	4 (2–6)	4 (2–6)	0.93 [0.89–0.96]^**1**^	**<0.001**
Any ICH–n (%)	122/902 **(13.5)**	117/1300 **(9.0)**	156/1269 **(12.3)**	208/1383 **(15.0)**	189/1397 **(13.5)**	1.06 [0.998–1.13]^**1**^	**0.06**
Symptomatic ICH (ECASS-II)–n (%)	39/894 **(4.4)**	41/1286 **(3.2)**	47/1254 **(3.7)**	70/1367 **(5.1)**	61/1384 **(4.4)**	1.07 [0.97–1.19]^**1**^	**0.18**
In-hospital mortality–n (%)	125/888 **(14.1)**	238/1294 **(18.4)**	240/1256 **(19.1)**	275/1284 **(21.4)**	265/1273 **(20.8)**	1.11 [1.04–1.17]^**1**^	**<0.01**
Early neurologic deterioration (worsening of NIHSS ≥4 at 24 h) – n (%)	169/848 **(19.9)**	244/1234 **(19.8)**	249/1174 **(21.2)**	284/1302 **(21.8)**	303/1298 **(23.3)**	1.08 [1.02–1.14]^**1**^	**<0.01**
Early neurologic improvement (∆NIHSS ≥4 or NIHSS = 0 at 24 h) – n (%)	369/848 **(43.5)**	520/1234 **(42.1)**	462/1174 **(39.4)**	494/1302 **(37.9)**	494/1298 **(38.1)**	0.95 [0.93–0.995]^**1**^	**0.03**
Successful recanalisation (mTICI 2b/3) – n (%)	742/884 **(83.9)**	1058/1275 **(83.0)**	1077/1241 **(86.8)**	1158/1364 **(84.9)**	1102/1289 **(85.5)**	1.07 [1.01–1.13]^**2**^	**0.01**
Complete recanalisation (mTICI 3)–n (%)	413/884 **(46.7)**	675/1275 **(52.9)**	686/1241 **(55.3)**	747/1364 **(54.8)**	698/1289 **(54.2)**	1.07 [1.03–1.11]^**2**^	**<0.001**
Successful recanalisation at first pass–n (%)	288/804 **(35.8)**	470/1190 **(39.5)**	474/1205 **(39.3)**	546/1337 **(40.8)**	531/1276 **(41.6)**	1.06 [1.01–1.10]^**2**^	**<0.01**
Vasospasm during procedure–n (%)	23/902 **(2.5)**	42/1300 **(3.2)**	92/1269 **(7.2)**	110/1383 **(8.0)**	96/1397 **(6.9)**	1.23 [1.06–1.27]^**3**^	**<0.01**
Dissection/perforation during procedure–n (%)	31/902 **(3.4)**	43/1300 **(3.3)**	40/1269 **(3.2)**	58/1383 **(4.2)**	47/1397 **(3.4)**	1.05 [0.95–1.17]^**3**^	**0.34**
Clot migration/Embolisation to the new territory during the procedure–n (%)	36/902 **(4.0)**	65/1300 **(5.0)**	52/1269 **(4.1)**	73/1383 **(5.3)**	68/1397 **(4.9)**	1.05 [0.96–1.15]^**3**^	**0.29**

1 Adjusted for age, sex, NIHSS at baseline, pre-stroke dependency (premorbid mRS >2), thrombolysis, time from last-seen well (or symptom onset) to hospital admission, diabetes mellitus, smoking status, hyperlipidaemia, arterial hypertension, atrial fibrillation, antiplatelet, anticoagulation, occlusion site (LVO vs. MeVo), successful recanalisation (mTICI 2b/3), centre.

2 Adjusted for thrombolysis, stroke aetiology (large-artery-atherosclerosis vs. else), occlusion site (MeVo vs. LVO vs. isolated extracranial [ordinal]), centre.

3 Adjusted for thrombolysis, stroke aetiology (large-artery-atherosclerosis vs. else), occlusion site (isolated extracranial vs. LVO vs. MeVO), number of passes, centre. Percentages and *p*-values <0.05 marked bold.

### Sensitivity analyses

For sensitivity analyses, our patient population was stratified by time from LSW to hospital arrival (≤6 h vs. >6 h). While the increase of mortality was most prominent in patients within the extended time window (from 28.2 to 39.2%; aOR 1.11 [1.00–1.24], *p* = 0.05), the results were consistent in patients presenting less than 6 h after LSW (from 25.4 to 32.8%, aOR 1.13 [1.06–1.20], *p* < 0.001). Except for a decrease in fair outcome (mRS ≤ 3), which was not present in the extended time window, all other clinical outcomes were consistent across time-to-treatment subgroups (LSW to admission >6 h vs. ≤ 6 h; see Supplementary Table S1). In a second sensitivity analysis, clinical outcomes stratified by pre-stroke disability (mRS > 2; y/n) were assessed. The increase of in-hospital complications such as END, any ICH, and in-hospital mortality was found to be most pronounced in patients with pre-stroke disability (see Supplementary Table S2). When comparing pre-pandemic (2017–2019) and pandemic (2020–2021) years, the pandemic time period was associated with worse outcomes (see Supplementary Table S3). However, actual COVID-19 rates were very low (*n* = 54, 1.9%), and in multivariable models, COVID-19 infection was not associated with any clinical outcome.

## Discussion

In this study, we present results from a large, prospectively collected national multicentre cohort of patients undergoing EVT at both academic and non-academic hospitals, reflecting clinical practise in experienced, high-volume centres in Germany. Over the 5-year period from 2017 to 2021, several important changes were observed: First, baseline characteristics shifted, with patients tending to be older, presenting with less severe strokes, and more frequently exhibiting medium vessel occlusions. Second, time metrics, diagnostic approaches, and treatment procedures evolved towards longer time from last seen well to hospital arrival, increased use of perfusion-based imaging, and decreased administration of intravenous thrombolysis. Third and most importantly, overall clinical outcomes deteriorated over time, primarily driven by an increase in mortality.

### Baseline characteristics

Between 2017 and 2021, we noticed a slight increase in age in our study population. This finding aligns with data from the landmark RCTs that established EVT in clinical practise. Earlier trials reported a median age of 68 years (HERMES meta-analysis), compared to a median/mean age between 69 and 71 years in the control and intervention groups of DAWN and DEFUSE-III (1, 3, 4). Chronological age, while often used as a variable to guide treatment decisions, may be seen as arbitrary to indicate or withhold EVT. Over time, increasing experience in EVT procedures and growing confidence in their safety and efficacy may have led physicians to expand the indication for EVT to include older patients, which could explain this trend in our data. Regarding stroke severity, the median NIHSS at admission declined from 15 to 13 points. Consistently, the target vessel occlusion site changed with a shift towards more medium vessel occlusions, foremost the MCA M2 segment. While EVT in MeVO seems to be a promising approach and may be beneficial, RCTs on EVT in MeVO are still ongoing, and robust evidence is lacking (8). In view of this, it is noteworthy that the proportion of MeVO patients among all EVT-treated patients in this study nearly doubled between 2017 and 2021, indicating that neurologists and neurointerventionalists seem to believe in the potential benefits of EVT in MeVO patients.

### Treatment times and intrahospital procedures

While the time from last seen well to hospital arrival remained stable at about 360 min in 2017 and 2018, it increased by more than 60 min in the following 3 years, reaching a median of 427 min in 2021. Similarly, the rate of patients presenting more than 6 h after stroke onset rose from 22.0 to 28.3%. This trend likely reflects the impact of new evidence from the DAWN and DEFUSE-III (3, 4) trials, which appear to have been integrated into clinical practise. In parallel with the extension of time-to-treatment, the use of perfusion-based imaging had a *relative* increase of approximately 50% in our study. At the same time, thrombolysis rates decreased from 52 to 40%. The latter may be due to multiple reasons: (1) higher rates of oral anticoagulation at baseline, (2) the growing proportion of patients presenting with unknown symptom onset or LSW exceeding 4.5 h, and (3) ongoing discussion about risks and benefits of bridging thrombolysis during the study period (7).

Notably, both the time from hospital admission to IVT bolus and the time from door-to-groin puncture increased during the study period. Several factors may explain these delays. First, the rising use of advanced imaging could have prolonged workflows and delayed EVT treatment decisions. Second, the increased rate of general anaesthesia may have been a crucial factor in longer door-to-groin times (19). Third, the ongoing debate regarding the risks and benefits of bridging thrombolysis may have led to discussions among the treating physicians, potentially delaying decision-making processes. Finally, while the SARS-COVID-19 pandemic was considered as a fourth possible contributing factor, previous analyses of our study population (GSR-ET registry) found no significant delays in in-hospital workflows during the COVID pandemic (20).

In 2017, evidence on general anaesthesia in EVT was scarce, yet secondary analyses from the HERMES collaboration suggested that the use of GA might be associated with worse clinical outcomes (21). Thus, it may be surprising that GA rates increased from 61.5 to 73.8% in the present study. Most probably, a meta-analysis of three randomised trials *directly* comparing GA to conscious sedation (published in 2019), which reported better clinical outcomes following GA (22), has contributed to this trend.

In the years preceding our study, substantial technical advances were made, with successful recanalisation of 57.3% of patients in MERCI (23), 71% in the trials pooled in HERMES (1), 76% in DEFUSE-3 (3), and 84% in DAWN (4). With reperfusion rates rising from 83.9 to 85.5%, the present study appears to build upon this development and indicates even further technical advances during recent years. Additionally, the strong association of GA with successful recanalisation, which has been reported in literature (22, 24), may further contribute to the increased technical success we found. Given that mTICI3 is associated with better outcomes than mTICI2b (25), together with the well-known impact of the first pass effect (26), it is encouraging that successful reperfusion at first pass changed from 35.8 to 41.6%, and the rate of mTICI3 increased from 46.7 to 54.2% in our study. Consistent with these findings, technical EVT success has continuously increased between 2015 and 2022 in a large French EVT registry (27). The present study and the aforementioned French study reflect the continuous advancement of both technical devices and, even more importantly, the growing expertise of neurointerventionalists.

### Clinical outcomes

At 3 months, about one in three patients reached functional independence (mRS ≤ 2), with minor yet statistically significant changes between 2017 and 2021. Ordinal mRS shift analysis indicated a significant trend towards worse functional outcomes over the 5-year period, with a salient decline between 2018 and 2019. This finding is consistent with data from the large multicentre ETIS registry in France, where clinical outcomes likewise deteriorated during these years (27). In our study, the worsening of clinical outcomes was predominantly due to a rise in mortality at 3 months from 25.3% (2017) to 34.7% (2021). Considering the lower NIHSS at admission, the higher proportion of MeVOs, and the increase in technical EVT success, this finding must be considered surprising. While the observed increase in age, decrease in IVT, and extended time-to-treatment may explain our findings in part, *year of treatment* remained significant after adjusting for these variables. We propose the following hypothesis: The slightly higher age may have been associated with higher covert pre-stroke morbidity, not necessarily transferring to pre-stroke dependency and, thus, unmeasured in our dataset. Higher rates of oral anticoagulation at admission may point to a relevant increase in cardiac conditions such as heart failure, which might have contributed to our results. Supporting the possible influence of pre-morbidity, our sensitivity analyses demonstrated a marked increase of END, any ICH, and in-hospital mortality between 2017 and 2021 in patients with pre-stroke disability (mRS > 2). We hypothesise that a higher disease burden may predispose these patients to the above-mentioned in-hospital complications. The increased END and its frequent occurrence may explain worsened clinical outcomes despite the lowered NIHSS at admission. However, since data on frailty, cognitive dysfunction, heart failure, or malignancy were not systematically collected, this hypothesis remains speculative. Moreover, the rigorous mismatch criteria of DAWN and DEFUSE might not have been strictly adhered to in clinical practise, possibly contributing to worse clinical outcomes in patients presenting in the extended time window. However, due to the lack of precise mismatch ratios in our data, we can only speculate on this matter. Given the crucial association of time-to-treatment with disability and mortality (28, 29), it may be expected that the continuous extension of LSW-to-reperfusion between 2017 and 2021 translates to increased mortality rates. Mortality after EVT in HERMES was similar to DEFUSE-III (15 and 14%, respectively) (1, 3). Mortality in DAWN, however, rose to 25% (4), which is consistent with our results and most probably due to the even longer extension of time-to-treatment in this trial.

Undeniably, the absolute mortality in our study exceeds the reported numbers in the above-mentioned RCTs. A previous analysis of the GSR-ET cohort, from which our study population derived, revealed that only a minority of patients in the registry met the inclusion criteria of milestone RCTs (11). This finding, together with the evident differences between RCTs and our study population regarding age, pre-stroke dependency, and morbidity, may provide a reasonable explanation for the higher absolute numbers of mortality. Since the SARS-CoV-2 pandemic began during our study period, the disruption in the healthcare system might have influenced clinical outcomes. Our sensitivity analyses, comparing the pre-COVID and COVID years, confirmed worse outcomes during the pandemic. However, the prevalence of COVID-19 infections in our study cohort was low (1.9%), and COVID-19 infection was not associated with any of the clinical outcomes in multivariable models. Consequently, it is unlikely that COVID-19 was a major factor in the increased mortality rate in our study population.

Considering the findings of the present study, it is important to note that there are no indicators of worse performance of EVT. On the contrary, technical outcomes indeed improved. We hypothesised that the worsened clinical outcomes may be more closely linked to changes in the patient population undergoing EVT, specifically longer time-to-treatment, higher age, and potentially increased general pre-morbidity.

Our study informs healthcare professionals, planners, and policymakers on the implementation of scientific evidence into clinical practise. As in any observational study, limitations have to be considered. First, our study did not include a control group of patients who did not undergo EVT. Thus, we were unable to assess trends regarding the efficacy of EVT. Second, our analysis is limited to data from experienced, high-volume neuro-interventional centres. Consequently, our results cannot be considered representative. Thirdly, despite providing a high rate of three-month follow-up (92%), we cannot exclude that missing data may have skewed our results (attrition bias). Fourth, ASPECTS at admission was available for only two-thirds of our study population. Thus, we did not include this variable in the statistical models. However, with no major changes in ASPECTS recorded over the 5-year period, this is unlikely to have introduced substantial bias.

Fifth, detailed results of perfusion-based imaging were not available, restraining adjustments for the extent of mismatch. Sixth, the exact vessel occlusion site in M2, ACA, and PCA was not recorded. Consequently, a differentiation between dominant and non-dominant M2 branches or ACA/PCA subsegments was not possible. Seventh, our study period did not exceed 2021. Thus, our data do not cover more recent developments, such as thrombectomy in large infarct core patients and the use of tenecteplase or thrombectomy of distal vessel occlusions (DiVo). Finally, all study data were provided by the participating sites, and the registry does not hold a central imaging reading. Thus, technical and clinical outcomes could not be adjudicated by independent investigators.

## Conclusion

Patients’ characteristics receiving EVT in experienced neurointerventional centres in Germany changed substantially between 2017 and 2021. Indication for EVT was expanded and included patients treated later and with medium vessel occlusions. Technical success rates improved. Higher rates of mortality may reflect a willingness to treat patients with more severe general health conditions.

## Data Availability

The raw data supporting the conclusions of this article will be made available by the authors, without undue reservation.

## German stroke registry endovascular treatment (GSR-ET) steering committee

Timo Uphaus (Chair; Uniklinik Mainz), Mario Abruscato (Klinikum Hanau), Jan Borggrefe (Mühlenkreiskliniken, Johannes Wesling Klinikum Minden), Michael Braun (Bezirkskrankenhaus Günzburg), Evdokia Evangelidou (Klinikum Nordstadt, Hannover), Bernd Eckert (Asklepios Klinik Altona, Hamburg), Ulrike Ernemann (Universitätsklinik Tübingen), Jens Fiehler (UKE Hamburg-Eppendorf), Fabian Flottmann (UKE Hamburg-Eppendorf), Arman Gregor (Klinikum Nordstadt, Hannover), Klaus Gröschel (Uniklinik Mainz), Gerhard F Hamann (Bezirkskrankenhaus Günzburg), Fee Keil (Uniklinik Frankfurt/ Main), Ilko L. Maier (Universitätsklinik Göttingen), Omid Nikoubashman (Universitätsmedizin Aachen), Sven Poli (Universitätsklinik Tübingen), Arno Reich (Universitätsmedizin Aachen), Joachim Röther (Asklepios Klinik Altona, Hamburg), Jan-Hendrik Schäfer (Uniklinik Frankfurt/Main), Maximilian Schell (UKE Hamburg-Eppendorf), Peter Schellinger (Mühlenkreiskliniken, Johannes Wesling Klinikum Minden), Götz Thomalla (UKE Hamburg-Eppendorf), Sven Thonke (Klinikum Hanau), Hanna Zimmermann [Uniklinik München (LMU)].
