# Supplementary material for: Time trends in mechanical thrombectomy (2017–2021): do real-world data reflect advances in evidence?
Source: Front Neurol. 2025 Feb 11;15:1517276. doi: 10.3389/fneur.2024.1517276 (PMC11850263; doi:10.3389/fneur.2024.1517276)
Supplement: Supplementary file 1 [file Table_1.DOCX]

**Supplement**

|  | **Table S1: Clinical Outcomes stratified by time-to-admission (≤6 hours vs. >6 hours)** | | | | | | | |
| --- | --- | --- | --- | --- | --- | --- | --- | --- |
|  |  | **2017**  **(n=902)*** | **2018**  **(n=1300)** | **2019**  **(n=1269)** | **2020**  **(n=1383)** | **2021**  **(n=1397)** | **aOR [95%-CI]**  **per +1 year** | **p** |
| mRS ≤ 2 at d90  – n (%) | **≤ 6 hours** | 234/610 **(38.4)** | 331/841 **(39.4)** | 285/762 **(37.4)** | 296/869 **(34.1)** | 290/754 **(38.5)** | 0.94  [0.89 – 1.00]^1^ | 0.049 |
|  | **> 6 hours** | 55/170 **(32.4)** | 86/271 **(31.7)** | 70/262 **(26.7)** | 98/328 **(29.9)** | 89/293 **(30.4)** | 0.95  [0.85 – 1.06]^1^ | 0.38 |
| mRS ≤ 3 at d90  – n (%) | **≤ 6 hours** | 324/610 **(53.1)** | 433/841 **(51.5)** | 381/762 **(50.0)** | 412/869 **(47.4)** | 365/754 **(48.4)** | 0.90  [0.85 – 0.95]^1^ | **<0.01** |
|  | **> 6 hours** | 70/170 **(41.2)** | 129/271 **(47.6)** | 99/262 **(37.8)** | 146/328 **(44.5)** | 125/293 **(42.7)** | 0.99  [0.90 – 1.10]^1^ | 0.90 |
| Mortality at d90  – n (%) | **≤ 6 hours** | 155/610  **(25.4)** | 230/849  **(27.3)** | 242/762  **(31.8)** | 270/869  **(31.1)** | 247/754  **(32.8)** | 1.13  [1.06 – 1.20]^1^ | **<0.001** |
|  | **> 6 hours** | 48/170  **(28.2)** | 85/271  **(31.4)** | 91/262  **(34.7)** | 105/328  **(32.0)** | 115/293  **(39.2)** | 1.11  [1.00 – 1.24]^1^ | 0.05 |
| mRS at d90  – median (IQR) | **≤ 6 hours** | 3 [1 – 6] | 3 [1 – 6] | 4 [ 1 – 6] | 4 [ 2 – 6] | 4 [ 1 – 6] | 0.92  [0.89 – 0.97]^1^ | **<0.001** |
|  | **> 6 hours** | 4 [2 – 6] | 4 [2 – 6] | 4 [2 – 6] | 4 [2 – 6] | 4 [2 – 6] | 0.94  [0.87 – 1.02]^1^ | 0.14 |
| Any ICH – n (%) | **≤ 6 hours** | 87/641 **(13.6)** | 78/884 **(8.8)** | 98/852 **(11.5)** | 124/903 **(13.7)** | 122/863 **(14.1)** | 1.08  [1.00 – 1.15]^1^ | 0.05 |
|  | **> 6 hours** | 26/179 **(14.5)** | 25/283 **(8.8)** | 39/292 **(13.4)** | 55/354 **(15.5)** | 38/339 **(11.2)** | 1.03  [0.90 – 1.16]^1^ | 0.69 |
| Symptomatic ICH (ECASS-II criteria) – n (%) | **≤ 6 hours** | 25/636 **(3.9)** | 29/876 **(3.3)** | 29/844 **(3.4)** | 41/893 **(4.6)** | 41/856 **(4.8)** | 1.09  [0.97 – 1.23]^1^ | 0.16 |
|  | **> 6 hours** | 9/177 **(5.1)** | 7/280 **(2.5)** | 10/286 **(3.5)** | 18/352 **(5.1)** | 12/339 **(3.5)** | 1.05  [0.85 – 1.30]^1^ | 0.64 |
| In-hospital mortality – n (%) | **≤ 6 hours** | 90/632 **(14.2)** | 150/878 **(17.1)** | 159/842 **(18.9)** | 172/828 **(20.8)** | 149/771 **(19.3)** | 1.11  [1.04 – 1.19]^1^ | **<0.01** |
|  | **> 6 hours** | 25/176 **(14.2)** | 59/283 **(20.8)** | 56/292 **(19.2)** | 71/335 **(21.2)** | 71/325 **(21.8)** | 1.11  [0.98 – 1.25]^1^ | 0.09 |
| Early neurologic improvement  (∆NIHSS ≥4 or NIHSS=0 at 24h)  – n (%) | **≤ 6 hours** | 287/605 **(47.4)** | 445/395 **(53.0)** | 346/898 **(43.3)** | 367/851 **(43.1)** | 359/820 **(43.8)** | 0.95  [0.90 – 0.998]^1^ | 0.04 |
|  | **> 6 hours** | 53/169 **(31.4)** | 92/274 **(33.6)** | 87/263 **(33.1)** | 100/338 **(29.6)** | 90/310 **(29.0)** | 0.98  [0.89 – 1.07]^1^ | 0.63 |
| Early neurologic deterioration (worsening of NIHSS ≥ 4 at 24h)  – n (%) | **≤ 6 hours** | 108/605 **(17.9)** | 143/840 **(17.0)** | 163/798 **(20.4)** | 172/851 **(20.2)** | 170/820 **(20.7)** | 1.08  [1.02 – 1.16]^1^ | 0.01 |
|  | **> 6 hours** | 44/169 **(26.0)** | 65/274 **(23.7)** | 52/263 **(19.8)** | 80/3338 **(23.7)** | 83/310 **(26.8)** | 1.05  [0.95 – 1.17]^1^ | 0.34 |

^1^adjusted for age, sex, NIHSS at baseline, pre-stroke-dependency (premorbid mRS >2), thrombolysis, time from last seen well (or symptom onset) to hospital admission, diabetes mellitus, smoking status, hyperlipidemia, arterial hypertension, atrial fibrillation, antiplatelet, anticoagulation, occlusion site (LVO vs. MeVo), successful recanalization (mTICI 2b/3), center

|  | **Table S2: Clinical Outcomes stratified by pre-stroke disability (mRS ≤ 2 vs. mRS > 2)** | | | | | | | |
| --- | --- | --- | --- | --- | --- | --- | --- | --- |
|  | **Pre-stroke** | **2017**  **(n=902)*** | **2018**  **(n=1300)** | **2019**  **(n=1269)** | **2020**  **(n=1383)** | **2021**  **(n=1397)** | **aOR [95%-CI]**  **per +1 year** | **p** |
| mRS ≤ 2 at d90  – n (%) | **No Disability** | 303/755 **(40.1)** | 446/1046 **(42.6)** | 371/968 **(38.3)** | 420/1112 **(37.8)** | 420/1054 **(39.8)** | 0.94  [0.89 – 0.99] | 0.03 |
|  | **Disability** | - | - | - | - | - |  |  |
| mRS ≤ 3 at d90  – n (%) | **No Disability** | 403/755 **(53.4)** | 583/1046 **(55.7)** | 491/968 **(50.7)** | 568/1112 **(51.1)** | 545/1054 **(51.7)** | 0.92  [0.87 – 0.97] | <0.01 |
|  | **Disability** | 20/97 **(20.6%)** | 21/183 **(11.5%)** | 21/150 **(14.0%)** | 35/186 **(18.8)** | 13/154 **(8.4)** | 0.95  [0.79 – 1.14] | 0.61 |
| Mortality at d90  – n (%) | **No Disability** | 165/755 **(21.9)** | 255/1046 **(24.4)** | 280/968 **(28.9)** | 309/1112 **(27.8)** | 315/1054 **(29.9)** | 1.12  [1.06 – 1.19) | <0.001 |
|  | **Disability** | 51/97 **(52.6)** | 97/183 **(53.0)** | 89/150 **(59.3)** | 97/186 **(52.5)** | 99/154 **(64.3)** | 1.13  [0.99 – 1.29] | 0.06 |
| mRS at d90  – median (IQR) | **No Disability** | 3 (1 – 5) | 3 (1 – 5) | 3 (1 – 6) | 3 (1 – 6) | 3 (1 – 6) | 0.93  [0.89 – 0.97] | <0.001 |
|  | **Disability** | 6 (4 – 6) | 6 (4 – 6) | 6 (4 – 6) | 6 (4 – 6) | 6 (4 – 6) | 0.91  [0.81 – 1.03] | 0.14 |
| Any ICH – n (%) | **No Disability** | 111/789 **(14.1)** | 102/1098 **(9.3)** | 135/1082 **(12.5)** | 169/1172 **(14.4)** | 163/1199 **(13.6)** | 1.04  [0.98 – 1.12] | 0.20 |
|  | **Disability** | 11/105 **(10.5)** | 15/195 **(7.7)** | 19/169 **(11.2)** | 33/196 **(16.8)** | 24/179 **(13.4)** | 1.24  [1.03 – 1.50] | 0.02 |
| Symptomatic ICH (ECASS-II criteria) – n (%) | **No Disability** | 27/782 **(4.7)** | 36/1085 **(3.3)** | 40/1069 **(3.7)** | 57/1160 **(4.9)** | 51/1188 **(4.3)** | 1.04  [0.93 – 1.16] | 0.50 |
|  | **Disability** | 2/105 **(1.9)** | 5/194  **(2.6)** | 7/167 **(4.2)** | 11/194 **(5.7)** | 9/177 **(5.1)** | 1.22  [0.95 – 1.56] | 0.12 |
| In-hospital mortality – n (%) | **No Disability** | 100/782 **(12.8)** | 172/1093 **(15.7)** | 180/1076 **(16.7)** | 205/1075 **(19.1)** | 196/1078 **(18.2)** | 1.10  [1.03 – 1.18] | <0.01 |
|  | **Disability** | 24/101 **(23.8)** | 61/194 **(31.4)** | 51/166 **(30.7)** | 61/195 **(31.3)** | 59/179 **(33.0)** | 1.15  [0.999 –1.31] | 0.052 |

| Early neurologic improvement  (∆NIHSS ≥4 or NIHSS=0 at 24h)  – n (%) | **No Disability** | 329/748 **(44.0)** | 467/1046 **(44.6)** | 418/1010 **(41.4)** | 440/1109 **(39.7)** | 453/1125 **(40.3)** | 0.96  [0.92 – 1.01] | 0.13 |
| --- | --- | --- | --- | --- | --- | --- | --- | --- |
|  | **Disability** | 38/95 **(40.0)** | 53/185 **(28.6)** | 43/151 **(28.5)** | 53/185 **(28.6)** | 41/162 **(25.3)** | 0.87  [0.76 – 0.999] | 0.048 |
| Early neurologic deterioration (worsening of NIHSS ≥ 4 at 24h)  – n (%) | **No Disability** | 152/748 **(20.3)** | 197/1047 **(18.8)** | 200/1008 **(19.8)** | 230/1109 **(20.7)** | 250/1125 **(22.2)** | 1.06  [1.003 – 1.13] | 0.04 |
|  | **Disability** | 16/95 **(16.8)** | 45/185 **(24.3)** | 43/151 **(28.5)** | 52/185 **(28.1)** | 49/162 **(30.2)** | 1.14  [0.76 – 0.98] | 0.08 |

^1^adjusted for age, sex, NIHSS at baseline, pre-stroke-dependency (premorbid mRS >2), thrombolysis, time from last seen well (or symptom onset) to hospital admission, diabetes mellitus, smoking status, hyperlipidemia, arterial hypertension, atrial fibrillation, antiplatelet, anticoagulation, occlusion site (LVO vs. MeVo), successful recanalization (mTICI 2b/3), center

| **Table S3: Clinical Outcomes pre- and post COVID-19-Pandemic** | | | | |
| --- | --- | --- | --- | --- |
|  | **Pre-pandemic years (2017 – 2019)** | **Pandemic years**  **(2020 – 2021)** | **aOR [95%-CI]**  **(pandemic vs. pre-pandemic)** | **p** |
| mRS ≤ 2 at d90  – n (%) | 1137/3225 **(35.3)** | 850/2535 **(33.5)** | 0.85  [0.73 – 0.98] | 0.02 |
| mRS ≤ 3 at d90  – n (%) | 1544/3225 **(47.9)** | 1163/2535 **(45.9)** | 0.83  [0.73 – 0.96] | <0.01 |
| Mortality at d90  – n (%) | 954/3225 **(29.6)** | 844/2535 **(33.3)** | 1.27  [1.10 – 1.46] | <0.01 |
| mRS at d90  – median (IQR) | 4 [2 – 6] | 4 [2 – 6] | 0.85  [0.76 – 0.94] | <0.01 |
| Any ICH – n (%) | 395/3471 **(11.4)** | 397/2780 **(14.3)** | 1.22  [1.03 – 1.44] | 0.02 |
| Symptomatic ICH (ECASS-II criteria) – n (%) | 127/3434 **(3.7)** | 131/2751 **(4.8)** | 1.25  [0.94 – 1.66] | 0.12 |
| In-hospital mortality – n (%) | 603/3438 **(17.5)** | 540/2557 **(21.1)** | 1.29  [1.10 – 1.52] | <0.01 |
| Early neurologic improvement  (∆NIHSS ≥4 or  NIHSS=0 at 24h)  – n (%) | 1351/3256 **(41.5)** | 988/2600 **(38.0)** | 0.89  [0.79 – 1.004] | 0.057 |
| Early neurologic deterioration (worsening or NIHSS ≥ 4 at 24h)  – n (%) | 662/3254 **(20.3)** | 587/2600 **(22.6)** | 1.19  [1.02 – 1.38] | 0.02 |

^1^adjusted for age, sex, NIHSS at baseline, pre-stroke-dependency (premorbid mRS >2), thrombolysis, time from last seen well (or symptom onset) to hospital admission, diabetes mellitus, smoking status, hyperlipidemia, arterial hypertension, atrial fibrillation, antiplatelet, anticoagulation, occlusion site (LVO vs. MeVo), successful recanalization (mTICI 2b/3), center

SARS-COV-19 (yes vs. no) is non-significant in multivariable models for all outcomes in the years 2020-2021.
